# Genetic diversity between local landraces and current breeding lines of pepper in China

**DOI:** 10.1038/s41598-023-29716-4

**Published:** 2023-03-11

**Authors:** Guangjun Guo, Baogui Pan, Xixi Yi, Abid Khan, Xuemei Zhu, Wei Ge, Jinbing Liu, Weiping Diao, Shubin Wang

**Affiliations:** 1 Institute of Vegetable Crops, Jiangsu Academy of Agricultural Sciences, Jiangsu Key Laboratory for Horticultural Crop Genetic Improvement, Graduate T&R Base of Zhengzhou University, Nanjing, 210014 China; 2 Department of Horticulture, The University of Haripur, Haripur, 22620 Pakistan

**Keywords:** Plant breeding, Plant genetics, Plant sciences

## Abstract

Based on 22 qualitative traits, 13 quantitative traits, and 27 molecular markers (26 SSR and 1 InDel), in the current study we compared the diversity and population structure of 94 local landraces and 85 current breeding lines of pepper in China. The results showed that the Shannon Diversity indices of 9 qualitative traits and 8 quantitative traits in current breeding lines were greater than those of landraces, of which 11 were fruit organ-related traits. Compared with current breeding lines, the mean values of Gene Diversity index and Polymorphism Information content of local landraces were higher by 0.08 and 0.09, respectively. Population structure and phylogenetic tree analysis showed that the 179 germplasm resources could be divided into two taxa, dominated by local landraces and current breeding lines, respectively. The above results indicated that the diversity of quantitative traits of current breeding lines were higher than that of local landraces, especially traits related to fruit organs, but the genetic diversity based on molecular markers was lower than that of local landraces. Therefore, in the future breeding process, we should not only focus on the selection of target traits, but also strengthen the background selection based on molecular markers. Moreover, the genetic information of other domesticated species and wild species will be transferred to the breeding lines through interspecific crosses to expand the genetic background of the breeding material.

## Introduction

Pepper (*Capsicum* spp.) is an important vegetable crop and condiment widely grown in tropical and subtropical regions of the world. The genus *Capsicum* has 5 cultivated species, *C. annuum*, *C. frutescens*, *C. baccatum*, *C. chinense* and *C. pubescens*, and 33 wild species^1^, while its genome size ranges from 3.3 to 3.6 GB, the most complex and largest genome in the Solanaceae family^2,3^.

Pepper germplasm resources showed huge differences at the phenotypic, biochemical and DNA levels, leading to high genetic diversity in the germplasm resources^4–6^. To ascertain the origin and process of domestication, the scientists examined the genetic diversity and population structure of semi-wild *Capsicum* germplasm and domesticated varieties collected from Central America (from central Mexico to northwest Costa Rica)^7,8^. Using molecular markers such as Amplified Fragment Length Polymorphism (AFLP), Simple Sequence Repeats (SSR) and Single Nucleotide Polymorphism (SNP), the genetic diversity and population structure of large-scale germplasm materials were analyzed, and some core germplasm banks were constructed to further understand the genetic diversity of germplasm materials^9–12^. Numerous studies on the genetic diversity and structure of China's pepper germplasm resources, including those for particular regions^13–15^, specific uses or fruit shape of chili germplasm resources^16–19^, pepper germplasm resources introduced from abroad^20–22^, and core germplasm for breeding materials^23^ have also been published.

Pepper was introduced to China in the late sixteenth century, and the earliest record was found in Gao Lian’s "*Zun Sheng Ba Jian*" in 1591. Due to the differences in ecological conditions and dietary habits of various regions of China, a large number of local varieties of pepper have been formed in the long-term natural selection and artificial improvement process^24^. In China, from 1965 to 1985, research was carried out on the collection, identification and preservation of pepper germplasm resources. At present, more than 2000 pepper germplasm resources are available in the National Genebank of China, which mostly consists of local landraces and also includes a small number of foreign commercial varieties^12,25^.

The pepper varieties in China are dominated by *C. annuum* species, with a small amount of *C. chinense*. In 1972, breeders produced the first hybrid pepper variety^26^. Since then, Chinese breeders have developed a wide range of current breeding lines and hybrid varieties using classical breeding techniques like hybridization and backcrossing along with modern breeding technologies like molecular marker-assisted selection^27,28^. With the innovation of pepper breeding technologies, the development and promotion of pepper varieties has progressed a lot^29,30^. Pepper varieties have been improved for three to four generations in China. Currently, hybrids account for more than 95% of the total pepper production^27,28,31,32^. In the 1970s, our team at the Institute of Vegetable Crops, Jiangsu Academy of Agricultural Sciences bred the first hybrid variety of pepper 'Zaofeng No.1' in China by using the local landrace 'Nanjing Zao Jiao' and the foreign introduced germplasm 'German'. Since then, we obtained a large number of advanced generation inbred lines through genetic improvement of the progeny of local landraces and domestic and foreign commercial varieties. Over the past 50 years, we have bred the 'Su Jiao' series of varieties, which have been widely promoted and utilized in Jiangsu, Shandong, Anhui, Guangdong and other significant pepper production areas, directly reflecting the development trend of pepper breeding in China.

Breeders generally agree that the genetic diversity of current pepper breeding lines is decreasing, but the exact situation is unclear. The reason is that there are few comparative studies of genetic diversity between current breeding lines and former local landraces. In this study, 179 materials were selected as research materials, including 94 local landraces and 85 current breeding lines. The 179 materials were primary core germplasm collections that we identified by evaluating 26 SSR markers and 1 InDel marker on 697 accessions, including 334 local landraces and 363 current breeding lines (data unpublished). The 334 local landraces were introduced from the National Genebank of China and originated from different regions of China in the 1980s, which were highly representative of varieties and breeding lines used 40 years ago in China. The 363 current breeding lines were improved and purified by our team in the recent five years. Among them, some current breeding lines mainly came from the improvement and selection of local landraces, while other current breeding lines came from the isolation and purification of commercial varieties from various regions of China. Therefore, the current breeding lines basically represent the main types of breeding materials for the market of China in recent years. Based on 35 phenotypic traits, the 179 accessions in the core germplasm collections represented about 70% of the genetic information present in the 697 accessions evaluated initially^33^. For the research reported herein, we evaluated the genetic diversity of 94 local landraces and 85 current breeding lines based on their phenotypic and molecular marker polymorphism in order to explore the changes in genetic diversity of pepper breeding resources after 40 years of artificial improvement and selection. This study will serve as a useful reference for guiding the future improvement of pepper breeding lines and the breeding of new varieties.

## Material and methods

### Plant materials

In the previous study, a core collection of 179 materials according to SSR analysis was established based on 334 local landraces and 363 current breeding lines (data unpublished). The local landraces were collected in different regions of China before 1985, almost 40 years ago. In contrast, the current breeding lines are 8–10 generation inbred lines that were used for commercial breeding in the last 5 years. A small percentage of current breeding lines were improved based on local landraces and most of them were purified and improved based on market varieties. The core collection of 179 materials were used in the study, including 94 local landraces and 85 current breeding lines. The 94 local landraces, of which 85 accessions originated from 26 provinces or regions in China, and the remaining 9 accessions were introduced from abroad (Table 1). The 85 current breeding lines were belong to the pepper innovation team of the Institute of Vegetable Research, Jiangsu Academy of Agricultural Sciences.

**Table 1:** The accession number, variety name and origin of the 94 local landraces used in the research.

Serial no.	Accession	Plant name	Origin	Serial No	Accession	Plant name	Origin
GW003	V06C0012	Linquan Chi Da Jiao	Anhui, China	GW259	V06C0996	Yanqi Jian Jian Jiao	Xinjiang, China
GW006	V06C0031	Sai Jiao	Anhui, China	GW261	V06C1024	Dazhuang Chang Lazi	Yunnan, China
GW031	V06C0113	Da Haijiao	Sichuan, China	GW263	V06C1055	Lajiao	Jiangsu, China
GW038	V06C0154	Nanping Dunjiao Zhitianjiao	Fujian, China	GW273	V06C1066	Wan Xuan 1 Hao	Anhui, China
GW040	V06C0157	Zhukou Wangkeng Zhitianjiao	Fujian, China	GW278	V06C1094	Gangu Da Lajiao	Gansu, China
GW046	V06C0164	Tongan Chang Mijiao	Fujian, China	GW280	V06C1110	Huaxian Lajiao	Guangdong, China
GW060	V06C0244	Guankoutian Lajiao	Guizhou, China	GW284	V06C1113	Niujiao Jiao	Guangxi, China
GW062	V06C0257	Kaiyang Lajiao	Guizhou, China	GW289	V06C1123	Yangjiao Jiao	Guangxi, China
GW065	V06C0272	Xiao Zhuo Jiao Jiao	Guizhou, China	GW291	V06C1164	Wuse Jiao	Guizhou, China
GW066	V06C0274	Tian Zhu La jiao	Guizhou, China	GW295	V06C1185	Yangjiao Jiao	Hebei, China
GW072	V06C0299	Jianjiao	Hebei, China	GW296	V06C1220	Xi 12	Heilongjiang, China
GW073	V06C0302	Shizi Lajiao	Hebei, China	GW299	V06C1223	Teda Niujiao Jiao	Heilongjiang, China
GW082	V06C0360	Weixian Jian Lajiao	Hebei, China	GW301	V06C1227	Small Chilli	Russia
GW086	V06C0385	Yunyang Jiao	Henan, China	GW303	V06C1290	Lajiao	Hubei, China
GW090	V06C0404	Yanjin Tianjiao	Henan, China	GW309	V06C1291	Xiao Qi Zimei	Hubei, China
GW097	V06C0441	Xiao Lajiao	Heilongjiang, China	GW315	V06C1297	Changyang Caijiao	Hubei, China
GW098	V06C0445	Hajiao Yi Hao	Heilongjiang, China	GW323	V06C1309	Da Niujiao	Hubei, China
GW102	V06C0463	Xiao Qingjiao	Heilongjiang, China	GW329	V06C1390	Fu Di Zao	Hubei, China
GW104	V06C0465	Denglong Hongjiao	Heilongjiang, China	GW331	V06C1429	Cai Niujiao	Hubei, China
GW106	V06C0488	Ai Jiao Huang Lajiao	Hubei, China	GW332	V06C1449	Yangjiao Jiao	Hubei, China
GW107	V06C0490	Chaotianjiao	Hubei, China	GW342	V06C1493	Wan Lajiao	Sichuan, China
GW117	V06C0536	Jinjialing Da Lajiao	Hunan, China	GW349	V06C1523	Niujiao Jiao	Hubei, China
GW118	V06C0541	Shaxi Lajiao	Hunan, China	GW355	V06C1546	Yangjiao Jiao	Hubei, China
GW120	V06C0548	Da Yuanzhui Lajiao	Hunan, China	GW357	V06C1577	Cai Jiao	Hubei, China
GW129	V06C0603	Dunhua Sandao Jin	Jilin, China	GW360	V06C1581	Denglong Jiao	Sichuan, China
GW132	V06C0621	Nongan Jianjiao	Jilin, China	GW369	V06C1589	Chilli	Russia
GW141	V06C0652	Denglong Jiao	Jiangsu, China	GW382	V06C1591	XC-1–3	Jilin, China
GW142	V06C0656	Xiao Huang Ke Jiao	Jiangsu, China	GW385	V06C1601	Hybelle	America
GW143	V06C0671	84–1	Jiangsu, China	GW389	V06C1618	MSU216	America
GW154	V06C0686	Wuhu Tianjiao	Jiangsu, China	GW396	V06C1635	MSU128	America
GW160	V06C0694	Xiao Lajiao	Liaoning, China	GW404	V06C1672	Zao Feng	Inner Mongoria, China
GW169	V06C0696	Da Jianjiao	Liaoning, China	GW406	V06C1692	Lajiao	Shandong, China
GW180	V06C0703	Fushun Jiao	Liaoning, China	GW408	V06C1695	Lajiao	Shandong, China
GW183	V06C0754	Taosha Lajiao	Jiangxi, China	GW410	V06C1700	Yangjiao Jiao	Shandong, China
GW192	V06C0755	Fengcheng Leng Shui Jiao	Jiangxi, China	GW412	V06C1720	Shanghai Tianjiao	Shanghai, China
GW194	V06C0773	Xiao Wang Jiao	Jiangxi, China	GW414	V06C1740	Sweet pepper	Netherlands
GW208	V06C0781	Xiabu Yangjiao Jiao	Jiangxi, China	GW417	V06C1756	Expert 82–223	Romania
GW213	V06C0787	San Dao Jin	Inner Mongoria, China	GW421	V06C1763	PBC066	AVRDC
GW221	V06C0817	Ledou Xian Lazi	Qinghai, China	GW422	V06C1773	Hami Tu Lajiao	Xinjiang, China
GW225	V06C0846	Jimo Ma Zui Lajiao	Shandong, China	GW424	V06C1811	Da La	Yunnan, China
GW228	V06C0870	Zhu Zui Jiao	Shandong, China	GW428	V06C1864	Etkeze	Hungary
GW234	V06C0882	Yexian Yichuanling	Shandong, China	GW433	V06C1881	Chang Jian Lajiao	Tibet, China
GW237	V06C0888	Yutai Da Tianjiao	Shandong, China	GW439	V06C1892	Zhu Dachang Lajiao	Tibet, China
GW246	V06C0896	Yanzhou Denglong Pi	Shandong, China	GW446	2019P157	Zi Se Jiao	Shandong, China
GW247	V06C0934	San Dao Jin	Shanxi, China	GW447	2019P158	Cai Xing	Shandong, China
GW250	V06C0959	Dali Laoya Wo	Shaanxi, China	GW448	2019P159	Long Zhu Jiao	Shandong, China
GW258	V06C0970	Dai Huang Jiao	Tianjing, China	GW450	2019P162	Wu Cai Jiao	Shandong, China

### Phenotypic traits measurement

Six plants of each material were grown in green houses at Nanjing in 2021 and 2022 for phenotypic traits measurement. The 35 phenotypic traits that were investigated in this study included 22 qualitative traits and 13 quantitative traits. Pepper phenotypic traits investigations were carried out from April to July, referring to the methods of Li and Zhang^34^, and modifications were done appropriately according to the actual situation (Tables 2, 3).

**Table 2:** Distribution frequencies and Shannon Diversity indexes and their differences for 22 qualitative traits evaluated in 94 local landraces (LLR) and 85 current breeding lines (CBL) of pepper in China.

Qualitative trait	Population	Shannon diversity index	Trait value and its frequency (%)								
			1	2	3	4	5	6	7	8	9
Plant habit			Upright	Semi-upright	Prostrate						
	LLR	0.68	17.0%	76.6%	6.4%						
	CBL	0.66	11.8%	78.8%	9.4%						
	Difference	+ 0.02	+ 5.3%	− 2.2%	− 3.0%						
Stem color			Green	Anthocyanin coloration of nodes	Purple						
	LLR	0.70	63.8%	35.1%	1.1%						
	CBL	0.68	56.5%	43.5%	0.0%						
	Difference	+ 0.02	+ 7.4%	− 8.4%	+ 1.1%						
Stem pubescence			Absent	Weak	Medium	Strong					
	LLR	0.85	8.5%	72.3%	16.0%	3.2%					
	CBL	0.83	16.5%	71.8%	10.6%	1.2%					
	Difference	+ 0.02	− 8.0%	+ 0.6%	+ 5.4%	+ 2.0%					
Leaf shape			Broad elliptic	Ovate	Lanceolate						
	LLR	0.37	1.1%	89.4%	9.6%						
	CBL	0.49	1.2%	83.5%	15.3%						
	Difference	− 0.12	− 0.1%	+ 5.8%	− 5.7%						
Leaf color			Light green	Green	Dark green	Purple					
	LLR	0.34	0.0%	89.4%	10.6%	0.0%					
	CBL	0.32	1.2%	92.9%	4.7%	1.2%					
	Difference	+ 0.02	− 1.2%	− 3.6%	+ 5.9%	− 1.2%					
Leaf pubescence			Absent	Weak	Medium	Strong					
	LLR	0.77	41.5%	56.4%	2.1%	0.0%					
	CBL	0.58	72.9%	27.1%	0.0%	0.0%					
	Difference	+ 0.19	− 31.5%	+ 29.3%	+ 2.1%	0.0%					
Peduncle attitude			Semi-drooping	Drooping	Erect						
	LLR	1.04	19.1%	44.7%	36.2%						
	CBL	1.07	22.4%	36.5%	41.2%						
	Difference	− 0.02	− 3.2%	+ 8.2%	− 5.0%						
Corolla color			White	Light green	Light purple	Purple					
	LLR	0.26	94.7%	1.1%	2.1%	2.1%					
	CBL	0.06	98.8%	1.2%	0.0%	0.0%					
	Difference	+ 0.20	− 4.1%	− 0.1%	+ 2.1%	+ 2.1%					
Anther color			Yellow	Blue	Blue-violet	Purple					
	LLR	0.99	2.1%	45.7%	44.7%	7.4%					
	CBL	0.99	5.9%	40.0%	52.9%	1.2%					
	Difference	0.00	− 3.8%	+ 5.7%	− 8.3%	+ 6.3%					
Stigma color			White	Light purple	Purple						
	LLR	0.47	87.2%	7.4%	5.3%						
	CBL	0.61	81.2%	11.8%	7.1%						
	Difference	− 0.14	+ 6.1%	− 4.3%	− 1.7%						
Stigma exertion			Shorter than stamens	Equal to stamens	Longer than stamens						
	LLR	0.65	3.2%	21.3%	75.5%						
	CBL	0.62	1.2%	24.7%	74.1%						
	Difference	+ 0.03	+ 2.0%	− 3.4%	+ 1.4%						
Fruit shape			Lantern	Cone	Ox-horn	Goat-horn	Slender	Helix	Finger	Spherical	Teardrop
	LLR	1.55	35.1%	5.3%	17.0%	33.0%	4.3%	1.1%	1.1%	2.1%	1.1%
	CBL	1.32	44.7%	2.4%	31.8%	4.7%	14.1%	2.4%	0.0%	0.0%	0.0%
	Difference	+ 0.23	− 9.6%	+ 3.0%	− 14.7%	+ 28.3%	− 9.9%	− 1.3%	+ 1.1%	+ 2.1%	+ 1.1%
Fruit color (before maturity)			Greenish white	Light yellow	Greenish yellow	light-green	Green	Dark green	Purple		
	LLR	1.31	3.2%	1.1%	3.2%	22.3%	50.0%	19.1%	1.1%		
	CBL	1.30	1.2%	0.0%	8.2%	15.3%	54.1%	17.6%	3.5%		
		+ 0.01	+ 2.0%	+ 1.1%	− 5.0%	+ 7.0%	− 4.1%	+ 1.5%	− 2.5%		
Fruit color (at maturity)			Yellow	Orange	Red	Dark red					
	LLR	0.44	2.1%	2.1%	89.4%	6.4%					
	CBL	0.52	5.9%	3.5%	87.1%	3.5%					
	Difference	− 0.08	− 3.8%	− 1.4%	+ 2.3%	+ 2.9%					
Depth of stalk cavity			Absent	Shallow	Medium	Deep					
	LLR	1.38	28.7%	20.2%	25.5%	25.5%					
	CBL	1.38	23.5%	27.1%	24.7%	24.7%					
	Difference	− 0.01	+ 5.2%	− 6.8%	+ 0.8%	+ 0.8%					
Fruit glossiness			Absent	Present							
	LLR	0.21	5.3%	94.7%							
	CBL	0.06	1.2%	98.8%							
	Difference	+ 0.14	+ 4.1%	− 4.1%							
Fruit texture of surface			Smooth	Slightly wrinkled	Medium wrinkled	Strongly wrinkled					
	LLR	1.06	54.3%	31.9%	9.6%	4.3%					
	CBL	1.09	38.8%	47.1%	8.2%	5.9%					
		− 0.04	+ 15.4%	− 15.1%	+ 1.3%	− 1.6%					
Fruit shoulder shape			Absent	Bulge	Slightly concave	Concave					
	LLR	1.23	13.8%	51.1%	19.1%	16.0%					
	CBL	1.28	12.9%	43.5%	17.6%	25.9%					
	Difference	− 0.06	+ 0.9%	+ 7.5%	+ 1.5%	− 9.9%					
Calyx aspect			Non-enveloping	Medium-enveloping	Enveloping						
	LLR	1.01	50.0%	33.0%	17.0%						
	CBL	0.95	57.6%	28.2%	14.1%						
	Difference	+ 0.06	− 7.6%	+ 4.7%	+ 2.9%						
Fruit shape of apex			Acute	Rounded	Depressed	Depressed with acute					
	LLR	1.04	56.4%	7.4%	30.9%	5.3%					
	CBL	1.17	45.9%	10.6%	35.3%	8.2%					
		− 0.13	+ 10.5%	− 3.1%	− 4.4%	− 2.9%					
Fruit navel appendages			Present	Absent							
	LLR	0.56	75.5%	24.5%							
	CBL	0.68	56.5%	43.5%							
	Difference	− 0.13	+ 19.1%	− 19.1%							
Fruit spiciness			Absent spicy	Mildly spicy	Medium spicy	Spicy					
	LLR	1.31	20.2%	42.6%	14.9%	22.3%					
	CBL	1.29	42.4%	27.1%	12.9%	17.6%					
	Difference	+ 0.02	− 22.1%	+ 15.5%	+ 2.0%	+ 4.7%

**Table 3:** Shannon Diversity indexes, coefficients of variation, and maximum, minimum, range, mean and standard deviation values and their differences for 13 quantitative traits evaluated in 94 local landrace (LLR) and 85 current breeding lines (CBL) of pepper in China.

Quantitative trait	Population	Diversity index	Coefficient of variation (%)	Max	Min	Range	Mean	SD
Plant height (cm)	LLR	2.04	22.3	168.3	38.3	130.0	112.5	25.0
	CBL	2.01	16.3	143.3	62.8	80.5	102.0	16.6
	Difference	+ 0.03	+ 6.0	+ 25.0	− 24.5	+ 49.5	+ 10.4	+ 8.4
Length of leaf blade (cm)	LLR	1.54	19.5	21.1	5.4	15.6	12.4	2.4
	CBL	2.04	26.6	26.4	7.9	18.5	16.4	4.4
	Difference	− 0.50	− 7.1	− 5.3	− 2.4	− 2.9	− 3.9	− 1.9
Width of leaf blade (cm)	LLR	1.70	23.6	10.6	2.5	8.2	5.6	1.3
	CBL	2.03	27.7	12.1	3.9	8.2	7.5	2.1
	Difference	− 0.34	− 4.1	− 1.5	− 1.5	0.0	− 1.9	− 0.8
Length of petiole (cm)	LLR	1.61	23.5	10.1	1.7	8.4	5.7	1.3
	CBL	2.04	29.1	13.7	3.5	10.2	8.0	2.3
	Difference	− 0.43	− 5.7	− 3.6	− 1.8	− 1.8	− 2.3	− 1.0
Date of the first flower (d)	LLR	1.93	13.0	124.4	73.6	50.9	92.3	12.0
	CBL	1.70	11.7	124.4	54.1	70.3	84.0	9.8
	Difference	+ 0.23	+ 1.3	0.0	+ 19.4	− 19.4	+ 8.2	+ 2.2
First flowing node	LLR	2.06	35.6	23.8	5.2	18.7	10.2	3.6
	CBL	1.75	22.9	16.3	4.5	11.8	9.7	2.2
	Difference	+ 0.31	+ 12.7	+ 7.5	+ 0.7	+ 6.8	+ 0.5	+ 1.4
Length of fruit pedicel (cm)	LLR	2.17	24.3	7.3	2.4	4.9	4.1	1.0
	CBL	2.04	27.6	9.0	2.6	6.4	5.0	1.4
	Difference	+ 0.13	− 3.3	− 1.6	− 0.2	− 1.4	− 0.9	− 0.4
Weight of single fruit (g)	LLR	0.98	97.8	202.1	1.1	200.9	37.8	37.0
	CBL	1.86	79.9	409.9	6.4	403.5	121.7	97.2
	Difference	− 0.88	+ 17.9	− 207.8	− 5.2	− 202.6	− 83.9	− 60.3
Longitudinal diameter of fruit (cm)	LLR	1.49	40.6	20.4	1.5	18.9	9.7	3.9
	CBL	1.87	36.2	36.4	7.4	29.1	18.5	6.7
	Difference	− 0.38	+ 4.3	− 16.1	− 5.9	− 10.2	− 8.9	− 2.8
Fruit transverse diameter (cm)	LLR	1.76	56.7	9.8	0.8	9.0	3.6	2.0
	CBL	1.90	51.2	11.3	1.1	10.1	5.0	2.6
	Difference	− 0.14	+ 5.6	− 1.4	− 0.3	− 1.1	− 1.4	− 0.5
Thickness of flesh (mm)	LLR	1.65	42.1	5.9	0.7	5.3	2.6	1.1
	CBL	2.01	43.6	9.1	1.4	7.7	3.9	1.7
	Difference	− 0.36	− 1.5	− 3.1	− 0.7	− 2.4	− 1.3	− 0.6
Number of locules	LLR	1.87	20.5	4.3	2.0	2.3	2.8	0.6
	CBL	1.70	18.4	4.3	2.0	2.3	3.2	0.6
	Difference	+ 0.17	+ 2.1	0.0	0.0	0.0	− 0.3	0.0
Fruit firmness (Pa)	LLR	1.73	50.0	89.4	11.9	77.5	30.5	15.3
	CBL	1.80	41.4	65.7	10.4	55.3	26.6	11.0
	Difference	− 0.07	+ 8.7	+ 23.7	+ 1.5	+ 22.2	+ 3.9	+ 4.3

### SSR analyses

The genomic DNA was extracted from young plantlets that had been selected from six individuals of each material by using the Cat#DP3112 kit (Beijing Bioteke Corporation). The quality and concentration of DNA was measured by 1% agarose gel electrophoresis and Nano Drop One (ThermoFisher Scientific, USA). The DNA concentrations were adjusted to 50 ng/μL and kept at − 20 °C in a refrigerator.

For SSR analysis, we used 26 microsatellite markers publicly available from Nicola et al. and 1 InDel from Guo et al. that spanned 12 pepper chromosomes^9,35^. Each SSR and InDel forward primer was labeled with one of four fluorescent dyes: 6-carboxy-fluorescein, 6-carboxy-tetramethylrhodamine, hexachloro-6-carboxy-fluorescein, or carboxy-X-rhodamine. All primers were synthesized by Tsingke Biotechnology Company (Nanjing, China).

PCR was performed in 10 μL reaction volumes containing PrimeSTAR Max Premix (2 ×) 5 μL (Takara, Beijing), 0.25 μL of each primer (10 µmol L^−1^), double distilled H_2_O 3.5 μL and approximately 1 μL of genomic DNA (50 ng/μL) as templates. Thermocycling was started at 98 °C for 3 min and followed by 30 cycles of 98 °C for 20 s, 55 °C for 10 s and 72 °C for 20 s, with a final extension at 72 °C for 5 min. Following the reaction, 2 μL of PCR product was taken for electrophoretic detection, and then the PCR product was diluted to 2 ng/μL. 10 μL of liz500 mixture (liz500:HiDi = 1:130) were added to each PCR product, which was then heated at 95 °C for 5 min and then cooled in ice water. Fluorescence detection was carried out on an ABI 3730 genetic analyzer (Applied Biosystems, USA).

### Data analysis

Phenotypic data were organized and summarized by using Microsoft Excel 2016 software. For qualitative traits, distribution frequencies were based on grading criteria. For quantitative traits, six plants or six fruits per material were evaluated, and results for each accession were given a score based on a scale of 1 to 10, according to the mean (M) and standard deviation (S) of the aggregate data. A score of 1 was given to any value less than M − 2S and a score of 10 was assigned to any value greater than or equal to M + 2S. Intermediate scores were based on increments of 0.5 S. Distribution frequencies were obtained from these scores. Shannon indices (H′) were also calculated from the scores (H′ = − ΣPiLnPi, where Pi denotes the frequency of accessions with the particular trait score), as was coefficient of variation (CV = SD/M × 100%, where SD is the standard deviation and M is the mean value.)

GeneMapper 4.0 software was used to evaluate the sizes of amplified fragments (SSR and InDel alleles) from different samples. The number of unique alleles, gene diversity index, heterozygosity, and polymorphism information content (PIC) were calculated using Power Marker v3.25 software. Genetic distances were calculated by the method of Cavalli-Sforza and Edwards (1967)^36^ and were used to construct a phylogenetic tree based on the unweighted pair-group method of mathematical averages (UPGMA 5.10 Software) as implemented in MEGA (Molecular Evolutionary Genetics Analysis) software.

To infer the population structure of the 179 *C. annuum* accessions from China, we used the model-based program STRUCTURE v2.3.4. The optimal number of genetic groups (K) were computed by performing eight Markov chain Monte Carlo runs for each value of K from 1 to 10. Each run consisted of 100,000 iterations, with a burn-in period of 30,000 iterations, and used an admixture model allowing for Hardy–Weinberg equilibrium, correlated allele frequencies, and independent loci. A Pr(X|K) index with respect to each K was used to calculate ΔK (Evanno et al. 2005) where X denotes the genotypes of the sampled individuals^37^. According to this approach, the optimal K is estimated as the location of the first peak of A ΔK =|L^2^(K)|/s[Pr(x|k)], where |L^2^(K)| denotes the absolute value of the second order rate of change of Pr(X|K), and s[Pr(x|k)] is the standard deviation of the Pr(X-|K), and s indicates standard deviation of L(K). The principal coordinate analysis (PCA) was performed with DARwin 5.0.158 Software.

### Ethics declarations

All plant experiments were carried out in accordance to relevant institutional, national, and international guidelines and legislation.

## Results

### Phenotypic diversity of qualitative traits

**Figure 1:**
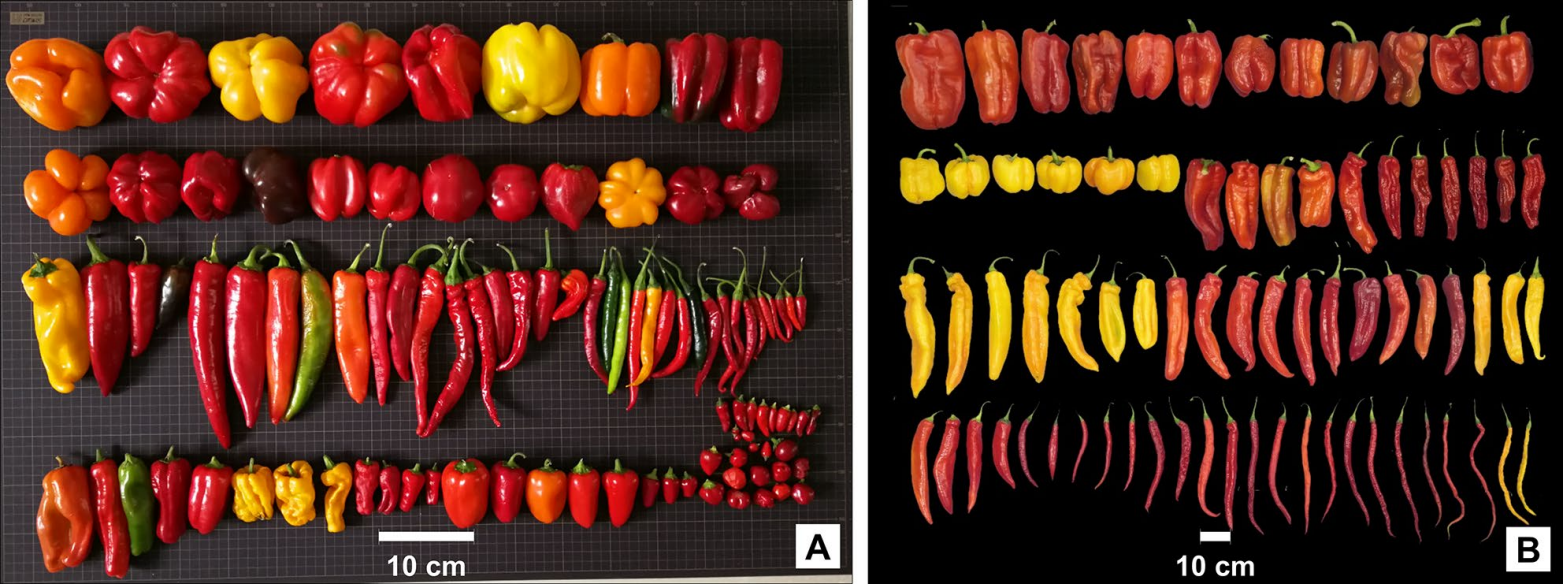
Examples of mature fruit types fruit harvested from the 94 local landraces (**A**) and from the 85 current breeding lines (**B**).

Twenty-two qualitative traits of 94 landraces and 85 current breeding lines were investigated, and the total number of variation types were 86, with a very rich variation in fruit morphology (Table 2 and Fig. 1). The Shannon Diversity indices of 22 qualitative traits of 94 landraces were between 0.21 and 1.55, with a mean value of 0.82. The Shannon Diversity indices of qualitative traits of 85 current breeding lines were between 0.06 and 1.39, with a mean value of 0.82. For overall comparison, the differences in Shannon Diversity indices for qualitative traits between local landraces and current breeding lines were not significant, indicating that the phenotypic diversity of the above-mentioned qualitative traits showed little difference between the two populations.

There were six qualitative traits related to plant morphology, including plant habit, main stem color, stem pubescence, leaf shape, leaf color and leaf pubescence (Table 2). The Shannon Diversity Index of stem pubescence was the greatest, but there was no significant difference between landraces and current breeding lines, while the Shannon Diversity Index of leaf pubescence had the greatest difference between the two populations. The Shannon Diversity index for leaf pubescence of the landraces was higher than that for the current breeding lines by 0.19. In current breeding lines, the proportion with no hairs on the leaf surface was 72.9%, which was 31.5% higher than that of local landraces, while the types with weak and medium hairs on the leaf surface was lower by 29.3% and 2.1%, respectively. The Shannon Diversity index of leaf shape was 0.12 higher in current breeding lines compared to local landraces. In local landraces and current breeding lines, the ovate leaf shape predominated, making up 89.4% and 83.5%, respectively. The frequency of lines with lanceolate leaf shape was higher by 5.7% in current breeding lines as compared to the local landraces. The Shannon Diversity indices of the other three traits were not significantly different between the two populations. (Table 2).

Five qualitative traits related to flowers, including peduncle attitude, corolla color, anther color, stigma color and stigma exertion were also examined (Table 2). The Shannon Diversity indices for corolla color were the lowest, but the greatest difference was found between the two populations, with local landraces being 0.20 higher than the current breeding lines. The percentage of lines with white corolla was 94.7% and 98.8% in the two populations. The percentage of lines with light purple or purple corolla was 0 and 4.2% in current breeding lines and local landraces, respectively. Secondly, the major difference between these two populations was found in the Shannon Diversity index for stigma color. The Shannon Diversity index of current breeding lines was 0.14 higher than that of the landraces. The white stigma was also the main trait, accounting for 87.2% and 81.2% of the landrace and current breeding lines, respectively. The proportion of light purple and purple stigmas in current breeding lines was higher by 3.4% and 1.4%, respectively. These results indicated that corolla color and stigma color were not closely linked, but inherited independently. The Shannon Diversity indices of the other three traits, peduncle attitude, anther color and stigma exertion were higher than those for the above two traits, but there were no significant differences between the landraces and current breeding lines (Table 2).

A total of 11 qualitative traits were related to the fruits, among which the fruit shape had the highest number (9) of variation types (Table 2). The Shannon Diversity indices were also highest, which were 1.55 and 1.32 in landraces and current breeding lines, respectively, with the largest difference between them. For fruit shape, lantern shape was most frequent, with 35.1% and 44.7%, respectively. Ox’s horn shape and goat’s horn shape had 17.0% and 31.8% in landraces, but 33.0% and 4.7% in current breeding lines, respectively. The proportion of linear shape peppers in current breeding lines was higher by 9.9% compared with that in landraces. In the current breeding lines, fruit morphologies other than spherical, teardrop, and finger-shaped had been eliminated. The acute fruit apex comprised the largest proportion of fruit apex shapes, accounting for 56.4% and 45.9%, respectively. The frequency of acute apexes in current breeding lines were 10.5% lower, while correspondingly there was an increase of 3.1%, 4.4% and 2.9% in rounded, depressed and depressed with acute apexes, respectively, thus the Shannon Diversity index of the apex fruit shape in the current breeding lines was 0.13 greater than that of the landraces. The two forms of variation "present" and "absent" for the quality traits of fruit appearance were fruit glossiness and fruit navel appendages. Both of the quality traits were dominated by “present” in local landraces and current breeding lines. So, the Shannon Diversity indices for both traits were low, but differed significantly between the two populations. For fruit glossiness, glossy materials accounted for 98.8% of current breeding lines, which was 4.1% higher than that of landraces. The Shannon Diversity index of landraces was 0.14 higher than that of current breeding lines. Regarding fruit navel appendages, the percentage of current breeding lines without fruit navel appendages was 19.1% greater than that of the landraces, and the Shannon Diversity index was 0.13 greater compared to that of landraces (Table 2).

For the other seven fruit related traits, the Shannon Diversity index differences between two populations was not significant, but there were certain regularities in the frequency distribution of variation types (Table 2). In terms of fruit color at maturity, the proportion of lines with yellow and orange fruits increased 3.8% and 1.4% in the current breeding lines. For texture of fruit surface, the proportion of current breeding lines with slightly wrinkled fruit surface increased by 15.1%, while the proportion of smooth fruit surface decreased by 15.4%. In terms of fruit spiciness, the frequency of absent spicy lines in current breeding lines increased by 22.1%, while the proportions of mildly spicy, medium spicy and spicy lines all decreased accordingly. The above results showed that the proportion of colorful sweet pepper lines, large fruit lines, fruit surface with slightly winkled lines and absent spicy lines increased in current breeding lines (Table 2).

### Phenotypic diversity of quantitative traits

Thirteen quantitative traits of 94 landraces and 85 current breeding lines were investigated. The Shannon Diversity indices for the 13 quantitative traits in the 94 local landraces ranged from 0.98 to 2.17, with a mean value of 1.73, while the Shannon Diversity indices for these 13 quantitative traits of the 85 current breeding lines ranged from 1.70 to 2.04, with a mean value of 1.90. Overall, the Shannon Diversity indices of the current breeding lines were higher than those of the local landraces (Table 3).

Plant height is a very important quantitative trait for breeding lines. The Shannon Diversity indices of plant height were 2.04 and 2.01 for landraces and current breeding lines, respectively, with no significant differences between them. Plant height of the local landraces ranged from 38.3 to 168.3 cm, with a range of 130.0 cm and a mean of 112.5 cm. The plant height of current breeding lines ranged from 62.8 to 143.3 cm, with a range of 80.5 cm and a mean of 102.0 cm. As compared with the local landraces, the current breeding lines were on average 10.4 cm shorter than the landraces. The results showed that the plant height differences among landraces were very large, while the plant height differences among current breeding lines were less, as was the average plant height (Table 3).

Leaf morphology included three quantitative traits, including length of blade, width of blade and petiole length. Compared with landraces, the Shannon Diversity indices of the three traits of current breeding lines increased by 0.5, 0.34 and 0.43 respectively, and the coefficient of variation increased by 7.15%, 4.15% and 5.66% respectively. Comparing the mean values of the three traits in the two populations, it was found that the average length and width of blade in the current breeding lines increased by 3.91 cm and 1.88 cm, respectively, and the average petiole length increased by 2.25 cm (Table 3).

Data of first flower and the first flowering node are maturity-related traits. The Shannon Diversity indices of the two traits in landraces were 1.93 and 2.06, respectively, while those in current breeding lines were 1.70 and 1.75, a reduction of 0.23 and 0.31, respectively. Comparing the maximum, minimum, range and average values of the two traits in the two populations, it was found that the longest flowering period in the two populations were both 124.4 days and the shortest were 73.6 days and 54.1 days, respectively. The average time of beginning of flowering of the current breeding lines was 8.2 days less than that of the local landraces. The first flowering node of local landraces ranged from 5.2 to 23.8 with a mean value of 10.2, while the range of first flowering node of current breeding lines ranged from 4.5 to 16.3 with a mean value of 9.7 (Table 3). The above results showed that the flowering characteristics related to maturity varied greatly among landrace lines, while these characteristics for current breeding lines were earlier than those of the local landraces.

Seven quantitative traits related to fruit included fruit pedicel length, single fruit weight, fruit longitudinal diameter, fruit transverse diameter, thickness of flesh, number of locules and the fruit firmness (Table 3). Compared with local landraces, the Shannon Diversity indices of current breeding lines for single fruit weight, fruit longitudinal diameter, fruit transverse diameter and thickness of flesh increased by 0.88, 0.38, 0.14 and 0.36, respectively. The single fruit weight of local landraces ranged from 1.1 to 202.1 g, with a mean value of 37.8 g. The single fruit weight of current breeding lines ranged from 6.4 to 409.8 g, with an average value of 121.7 g. The mean value of single fruit weight of current breeding lines was 3.2 times higher than that of landraces. Fruit longitudinal diameter, transverse diameter and thickness of flesh were closely related to single fruit weight. The average values of the above three traits in current breeding lines increased by 8.9 cm, 1.4 cm and 0.13 cm, respectively. The Shannon Diversity indices of fruit pedicel length and number of locules in current breeding lines was less 0.13 and 0.17, respectively, compared to that of landraces. The fruit pedicel lengths of local landraces and current breeding lines ranged from 2.4 to 7.3 cm and 2.6 to 9.0 cm, respectively, with an average value of 4.1 cm and 5.0 cm, respectively. The average fruit pedicel length of current breeding lines was 0.9 cm longer than that of landraces. There was no difference in the range of the number of locules between landraces and current breeding lines, but the mean number of locules in landraces and current breeding lines were 2.8 and 3.2, respectively. This result may be related to the 9.6% increase in the proportion of lantern-shaped pepper in current breeding lines. Compared with landraces, the mean of fruit firmness was less 3.95 Pa in current breeding lines, which may be due to the higher proportion of lines with large fruit in current breeding lines (Table 3).

### Genetic diversity based on SSR and InDel markers

The genotyping of 94 landraces and 85 current breeding lines revealed 26 SSR and 1 InDel marker loci diversity (Tables 4, 5).

**Table 4:** Genetic diversity analysis of 94 local landraces at 26 polymorphic simple sequence repeat (SSR) loci and 1 insertion-deletion (InDel) loci.

Marker	Major.Allele.Frquency	GenotypeNo	SampleSize	No. of obs	AlleleNo	GeneDiversity	Heterozygosity	PIC
Hpms1-214	0.71	19	94	94	15	0.49	0.13	0.47
Gpms104	0.51	5	94	94	6	0.53	0.94	0.43
Epms331	0.22	31	94	94	20	0.88	0.22	0.87
Epms755	0.63	8	94	94	6	0.52	0.14	0.44
Epms397	0.24	24	94	94	14	0.86	0.21	0.84
Es350	0.41	10	94	94	6	0.72	0.09	0.67
Gpms6	0.72	9	94	94	10	0.45	0.45	0.42
InDel-3–5	0.53	14	94	94	10	0.68	0.13	0.66
Hpms1-111	0.51	13	94	94	6	0.67	0.24	0.64
Epms386	0.42	22	94	94	15	0.75	0.18	0.72
HpmsAT2-14	0.87	9	94	94	7	0.24	0.05	0.24
Epms391	0.91	3	94	94	2	0.16	0.02	0.14
Epms310	0.27	26	94	94	14	0.84	0.20	0.82
HpmsE095	0.89	12	94	94	10	0.21	0.09	0.21
Gpms101	0.75	5	94	94	4	0.38	0.07	0.33
Epms725	0.85	8	94	94	6	0.28	0.07	0.26
HpmsCaSIG19	0.58	12	94	94	7	0.61	0.14	0.58
Epms419	0.60	10	94	94	7	0.59	0.11	0.56
HpmsE008	0.44	13	94	94	8	0.72	0.13	0.68
HpmsE088	0.76	10	94	94	8	0.41	0.05	0.38
EPMS680	0.78	10	94	94	9	0.38	0.03	0.37
Epms342	0.27	27	94	94	19	0.86	0.17	0.85
GPMS29	0.31	18	94	94	8	0.79	0.17	0.76
Hpms2-24	0.28	15	94	94	9	0.81	0.16	0.79
HpmsE013	0.53	14	94	94	11	0.66	0.07	0.63
Gpms169	0.72	13	94	94	10	0.46	0.10	0.43
HpmsE128	0.82	5	94	94	4	0.30	0.01	0.27
Mean	0.58	13.5	94	94	9.3	0.56	0.16	0.53

**Table 5:** Genetic diversity analysis of 85 current breeding lines at 26 polymorphic simple sequence repeat (SSR) loci and 1 insertion-deletion (InDel) loci.

Marker	Major.Allele.Frquency	GenotypeNo	SampleSize	No. of obs	AlleleNo	GeneDiversity	Heterozygosity	PIC
Hpms1-214	0.35	8	85	85	7	0.72	0.01	0.67
Gpms104	0.49	7	85	85	8	0.57	0.93	0.47
Epms331	0.28	14	85	85	11	0.82	0.06	0.79
Epms755	0.66	3	85	85	2	0.45	0.02	0.35
Epms397	0.21	13	85	85	9	0.84	0.05	0.82
Es350	0.99	2	85	85	2	0.01	0.01	0.01
Gpms6	0.97	3	85	85	3	0.06	0.06	0.06
InDel-3–5	0.33	10	85	85	11	0.80	0.01	0.78
Hpms1-111	0.56	10	85	85	7	0.61	0.09	0.57
Epms386	0.58	7	85	85	7	0.59	0.01	0.55
HpmsAT2-14	0.95	4	85	85	3	0.09	0.02	0.09
Epms391	0.83	4	85	85	3	0.29	0.01	0.25
Epms310	0.50	10	85	85	9	0.69	0.01	0.66
HpmsE095	0.93	4	85	85	4	0.13	0.00	0.13
Gpms101	0.59	6	85	85	5	0.50	0.07	0.39
Epms725	0.98	3	85	85	3	0.03	0.01	0.03
HpmsCaSIG19	0.32	15	85	85	8	0.79	0.12	0.76
Epms419	0.66	9	85	85	6	0.52	0.05	0.48
HpmsE008	0.45	7	85	85	4	0.69	0.04	0.64
HpmsE088	0.59	9	85	85	9	0.60	0.00	0.57
EPMS680	0.85	4	85	85	4	0.27	0.00	0.26
Epms342	0.66	23	85	85	19	0.55	0.15	0.54
GPMS29	0.46	9	85	85	8	0.72	0.04	0.68
Hpms2-24	0.71	7	85	85	6	0.46	0.02	0.42
HpmsE013	0.71	6	85	85	6	0.47	0.00	0.43
Gpms169	0.81	7	85	85	7	0.33	0.01	0.32
HpmsE128	0.68	5	85	85	4	0.45	0.04	0.37
Mean	0.63	7.7	85	85	6.5	0.48	0.07	0.45

A total of 251 alleles were amplified from 27 markers in the landraces. The number of alleles at different loci ranged from 2 to 20, and the average number of alleles per locus was 9.3. Gene Diversity index (GDI) and Polymorphism Information content (PIC) ranged from 0.16 to 0.88 and 0.14 to 0.87, with an average value of 0.56 and 0.53, respectively. Among these markers, the GDI and PIC of marker Epms331 were the highest, which were 0.88 and 0.87, respectively. The GDI and PIC of marker Epms391 was the lowest, which were 0.16 and 0.14, respectively (Table 4 and Supplementary Fig. 1). Observed heterozygosity was highly variable between loci (0.01–0.94), with an average value of 0.16, indicating that the local landraces were not fixed to homozygosity (Table 4).

A total of 175 alleles were amplified from 27 markers in the current breeding lines, with a range of 2 to 19 alleles at different loci and an average number of 6.48 alleles per locus. The GDI and PIC varied from 0.01 to 0.84 and 0.01 to 0.82, respectively, with mean values of 0.48 and 0.45. The GDI and PIC of Epms397 were the highest, which were 0.84 and 0.82, respectively. The lowest GDI and PIC were found for marker Es350, both of which were 0.01 (Table 5 and Supplementary Fig. 2). The Heterozygosity ranged from 0.01 to 0.93, with an average value of 0.07 in the current breeding lines (Table 5).

### Population genetic structure and phylogenetic tree analysis

**Figure 2:**
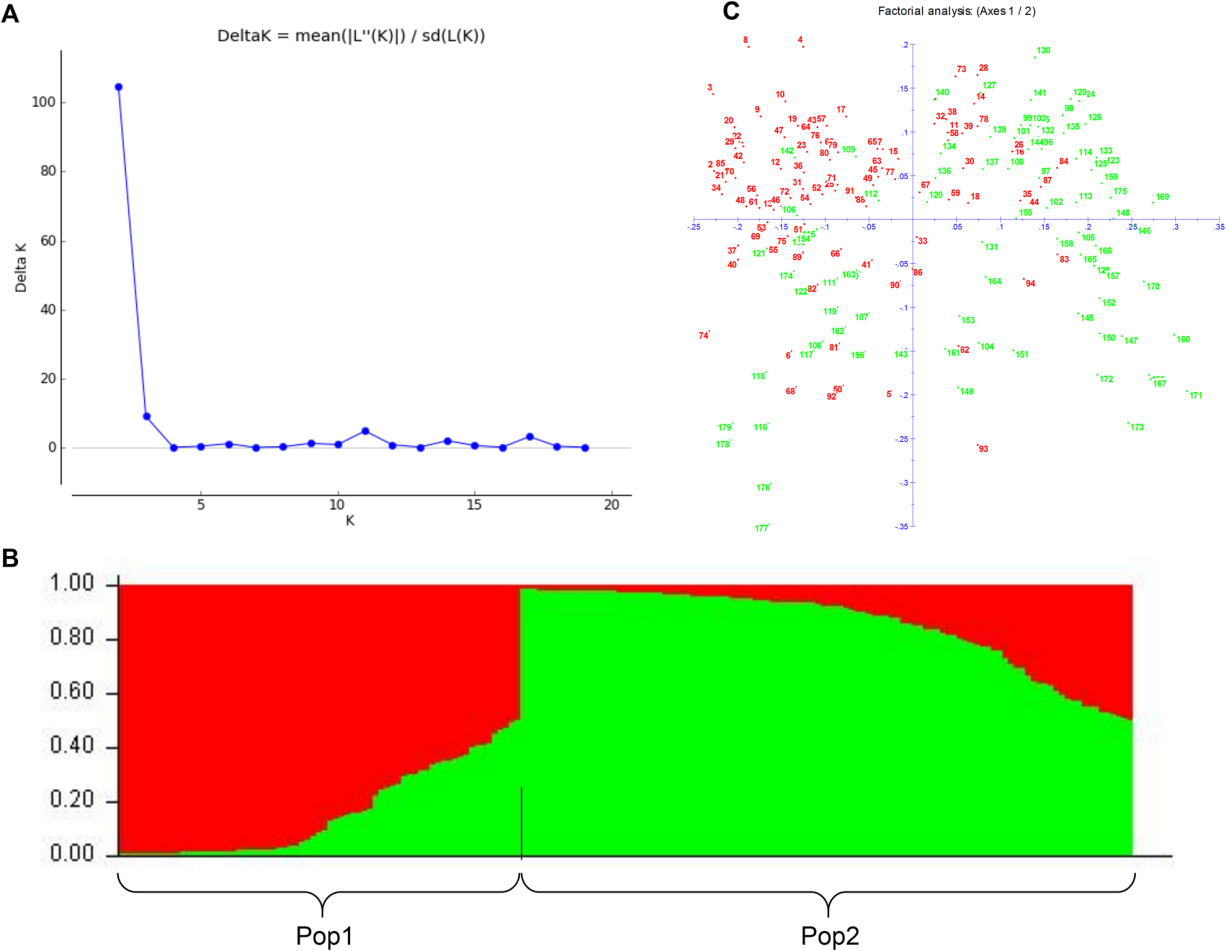
Results of population structure analysis based on molecular markers present in 94 Local landraces (green) and 85 current breeding lines (red) of pepper. (**A**) Distribution of ΔK as determined by eight Markov chain Monte Carlo runs. K, the optimal number of genetic groups. (**B**) Two pop inferred by genetic structure analysis. Red mean current breeding lines, Green mean local landraces. (**C**) Principal coordinate analysis of 179 accessions without missing simple sequence repeat (SSR) genotype data. In (**C**) red mean current breeding lines, green mean local landraces.

To investigate the differences of genetic background between 94 landraces and 85 current breeding lines, a population genetic structure analysis was performed by using the 27 markers. The ΔK analysis representing the population structure (Fig. 2A) showed that the ΔK value was maximum at K = 2, i.e., the 179 pepper lines could be divided into two groups, Pop1 and Pop2, where Pop1 contained 70 lines and Pop2 contained 109 lines (Fig. 2B).

The Pop1 group was predominated by current breeding lines, with 55 current breeding lines (78.5%), and 15 landraces (21.5%). The Pop2 group was predominated by landraces, with 79 local landraces (72.5%), and 30 current breeding lines, (27.5%). The number of lines with Q values greater than 0.6 in Pop1 and Pop 2 were 62 and 95, accounting for 88.6% and 87.2%, respectively, and 8 and 14 lines with Q values less than 0.6, respectively. The above results indicated that most of the Pop1 and Pop2 lines had a separate sources of genetic components, but some lines combined genetic components from both sources. There was some genetic information shared between the groups. The results for all 179 pepper lines were subjected to principal coordinate analysis, which supported the classification of the two groups, and some lines shared genetic components for both sources (Fig. 2C).

**Figure 3:**
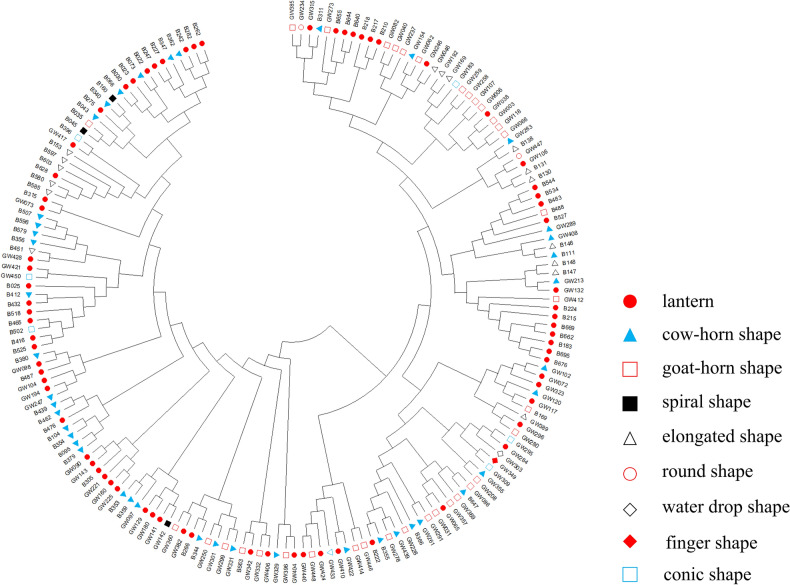
Phylogenetic tree of 94 local landraces (No. start with GW) and 85 current breeding lines (No. start with B) based on SSR and InDel markers using the NJ clustering method. (UPGMA 5.10, https://www.megasoftware.net/.

The genetic divergence of the 179 lines ranged from 0.04 to 0.90 with an average genetic distance of 0.50. The genetic distances between the GW422 and B644 lines were the most distant, while the genetic distances between the B507 and B596 lines were the closest. Based on the genetic distance, the Neighbor Joining (NJ) cluster tree was constructed. The results showed that the 179 lines could be divided into two clusters. Cluster I included 85 lines, including 54 current breeding lines and 31 local landraces, accounting for 63.53% and 36.47%, respectively. Cluster II included 94 lines, including 63 local landraces and 31 current breeding lines, accounting for 67.02% and 32.98%, respectively. In the clustering process, some lines with similar agronomic traits were clustered together, such as B227, B247, B262, and B282were all long lantern-shaped, thin-flesh and mildly spicy peppers, while B305, GW090, GW143, GW221, GW160, and GW225 were all long lantern-shaped and thick-flesh sweet peppers. Meanwhile, the ox-horn-shaped and goat-horn-shaped lines, which accounted for a high proportion, also had similar preferential clustering. However, some lines of different fruit shape were clustered together, probably because some of the current breeding lines were obtained by purification after crossing of different fruit shapes (Fig. 3).

## Discussion

In this study, the Shannon Diversity indices of qualitative traits of current breeding lines were higher for fruit shape, depth of stalk cavity, fruit color (before maturity) and fruit spiciness (Table 2). All the above four traits were associated with fruit organs, indicating that fruit diversity was abundant, which was consistent with the results of previous studies^20,22,38^. The Shannon Diversity indices of qualitative traits of current breeding lines were lower for corolla color, fruit glossiness and leaf color (Table 2), indicate that there was less genetic diversity. Compared to the Shannon Diversity indices of qualitative traits, the Shannon Diversity indices of quantitative traits was higher, implying more diversity of quantitative traits, (Tables 2, 3). This result was consistent with those of in previous reports^11,12^. Among the 13 quantitative traits, the Shannon Diversity indices of the length of fruit pedicel was highest, followed by the plant height (Table 3). The Shannon Diversity indices of the length of fruit pedicel in this study was higher than the results of the previous study^20,22,38^. The coefficients of variation for single fruit weight and fruit transverse diameter were over 50% (Table 3), indicating great variation among lines within the population.

Analysis of 22 qualitative traits in 94 local landraces and 85 current breeding lines revealed significant differences in Shannon Diversity indices of eight qualitative traits between the two populations. In comparison with local landraces, the Shannon Diversity indices of four qualitative traits, including leaf pubescence, corolla color, fruit shape, and fruit glossiness in current breeding lines were lower. While the Shannon Diversity indices for leaf shape, stigma color, fruit shape of apex, and fruit navel appendages were higher in current breeding lines (Table 2). Specifically, for each trait, the percentages of current breeding lines without leaf pubescence, glossy fruit surface, lanceolate leaf shape, light purple and purple stigma, rounded, depressed and depressed with acute of fruit apex and without fruit navel appendages increased by 31.5%, 4.1%, 5.7%, 6.0%, 10.5% and 19.1%, respectively. In contrast, current breeding lines with purple or light purple corollas and finger-shaped, spherical-shaped and teardrop-shaped fruits were likely discarded in the selection process. The above results indicated that the increase in the proportion of current breeding lines with lanceolate leaf shape and rounded, depressed and depressed with acute fruit apex reflected the diversification of current breeding lines, while the breeding process improved the commercial acceptance of fruit appearance by improving the fruit surface gloss and reducing the fruit navel appendages.

To summarize, for the flowering characteristics related to maturity, the Shannon Diversity indices of date of first flower and first flowering node in current breeding lines were less, with flowering time being earlier and first flowering node being lower. The above results indicated that the diversity of flowering characteristics in current breeding lines were reduced and flowering stages tended to be earlier. For leaf-related traits, the Shannon Diversity indices for length of blade, width of blade and petiole length of current breeding lines were higher, while the mean values were greater, 3.9 cm, 1.9 cm and 2.3 cm, respectively. Compared to local landraces, the Shannon Diversity indices for five of the seven fruit organ-related traits in current breeding lines were higher, indicating increased fruit diversity during artificial selection. We thought the main reason for this result might be that most of the current breeding lines were obtained by isolation, purification and improvement of market varieties, including all kinds of varieties from different regions of China. In addition, the diversity of cultivation facilities as well as consumption diversity in China may also influence the genetic diversity of current breeding lines. The aforementioned findings showed that in order to adjust to the facilitated cultivation conditions and market demand, breeders preferred the lines with large leaves, earlier flowering, lower flowering nodes, and higher single fruit quality during these election process. However, the increase of the proportion of large fruit lines likely led to an increase in the number of fruits locules and a decrease in fruit firmness.

Compared with the current breeding lines, the local landraces had 76 more alleles in 27 markers, with 2.8 more alleles per locus, and the mean values of GDI and PIC were 0.08 and 0.09 higher, respectively. In addition, the mean value of heterozygosity of 27 markers in the landraces was 0.16, while the heterozygosity of the current breeding lines was 0.07. The results indicated that some accessions of the local landraces were more Heterozygosity, while the purity of current breeding lines was higher than that of local landraces (Tables 4, 5). The above results indicated that the genetic diversity of local landraces was higher than that of current breeding lines, while the heterozygosity of current breeding lines was less than that of local landraces.

While using the same SSR molecular markers as used in this study, the germplasm resources used in the previous studies were more numerous and more broadly sourced compared to those used in the present study, so the GDI and PIC values were higher in those studies compared to those reported herein. For example, Nicola et al. analyzed 1352 pepper accessions using 28 SSR markers, and the mean values of the GDI and PIC were 0.70 and 0.67, respectively^9^. Zhang et al. analyzed 372 landraces of China by using 28 SSR markers, and the mean values of the GDI and PIC were 0.63 and 0.60, respectively^11^. However, Lee et al. analyzed 3821 accessions of 11 *Capsicum* species using 48 SNP markers, and the mean value of PIC was 0.38. Among them, 3383 *C. annuum* accessions had the highest PIC of 0.69^10^. Cheng et al. used 5149 SNP markers to analyze the genetic diversity of 398 accessions (local landraces and backbone parents) with GDI and PIC of 0.36 and 0.29, respectively^39^. Genetic diversity analysis of a 200-member core collection using 61 SSR markers showed the mean values of GDI and PIC were 0.40 and 0.35, respectively^23^. The above results indicated that the diversity of pepper accessions of China was lower compared with accessions from some other countries, and artificial selection has led to a further reduction in the genetic diversity of breeding lines.

The population genetic structure analysis and principal coordinate analysis indicated that there were relatively significant differences of the genetic backgrounds between the 94 local landraces and 85 current breeding lines, but some lines in the two populations likely shared genetic backgrounds. More similar to the results of the present study, Gu et al. clustered 1904 germplasm resources of China into two major taxa by population structure analysis and principal coordinate analysis^12^. Cheng et al. used 5149 SNP markers to classify 398 local landraces and backbone lines into two different taxa^39^. Meanwhile, phylogenetic tree analysis in this study showed that the clustering results based on genetic distance were correlated with fruit shape, and germplasms of the same fruit shape were more likely to be clustered together. This result was consistent with several previous reports^12,23^.

## Supplementary Information

Supplementary Figure 1.

Supplementary Figure 2.

Supplementary Table 1.

Supplementary Table 2.

Supplementary Legends.

## Data Availability

The 94 local landraces were conserved at the National Mid-term Genebank for Vegetables (https://ivf.caas.cn/jgsz/kybm/zzzyyjs/index.htm). The 85 current breeding lines were conserved at Jiangsu Academy of Agricultural Sciences. Information of breeding lines can be obtained by contacting Dr. Guo via email, ggj-198@163.com. We have obtained permission from the above institutions to use the 94 local landraces and the 85 current breeding lines in this study. The phenotypic data and genotype data of 94 local landraces and 85 current breeding lines of pepper are shown in Supplementary Table 1 and Supplementary Table 2, respectively.
