# Supplementary figures and images for: Genetic diversity between local landraces and current breeding lines of pepper in China
Source: Sci Rep. 2023 Mar 11;13:4058. doi: 10.1038/s41598-023-29716-4 (PMC10008637; doi:10.1038/s41598-023-29716-4)

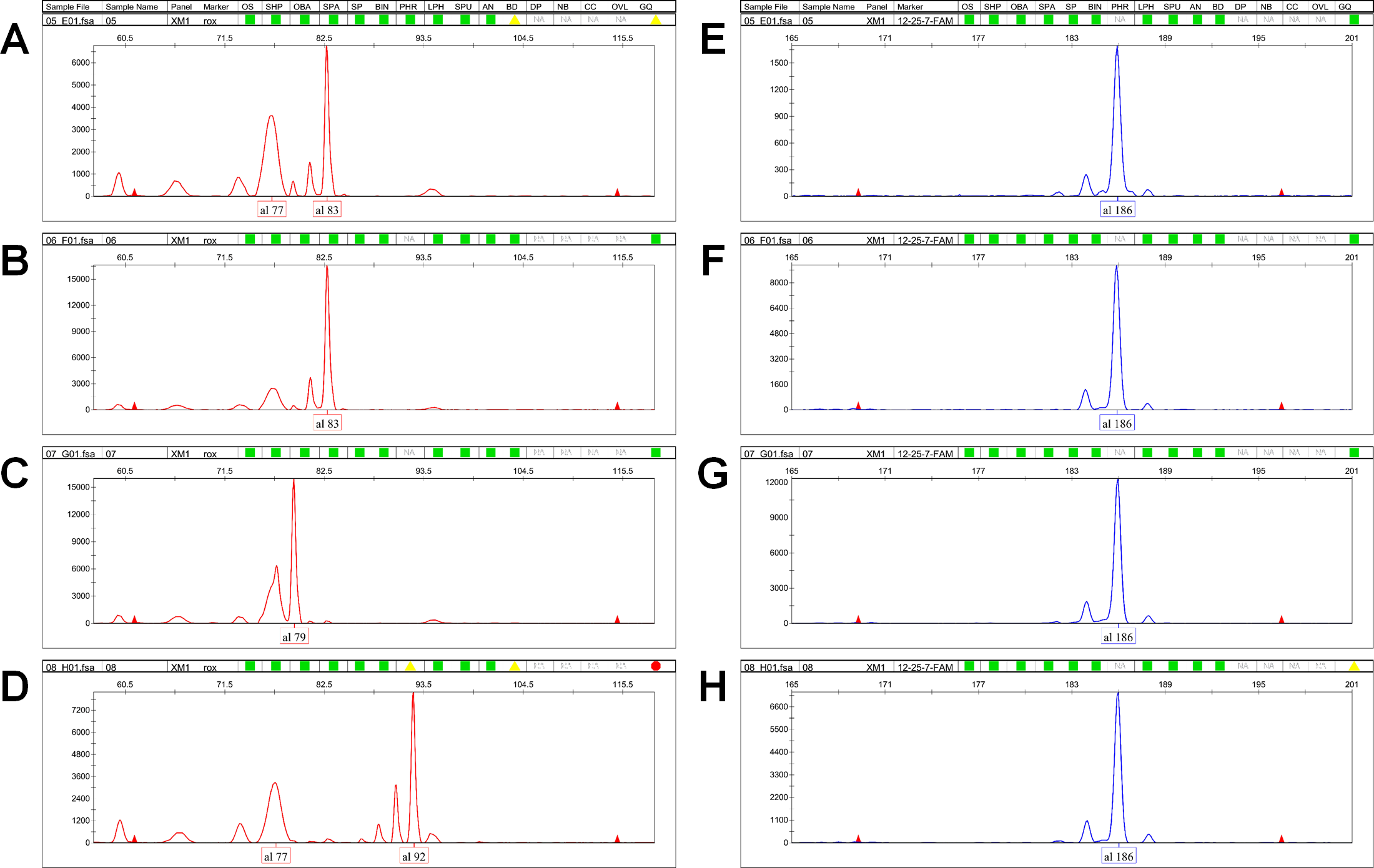

Supplement: Supplementary file 1 — Supplementary Figure 1. [file 41598_2023_29716_MOESM1_ESM.tif]

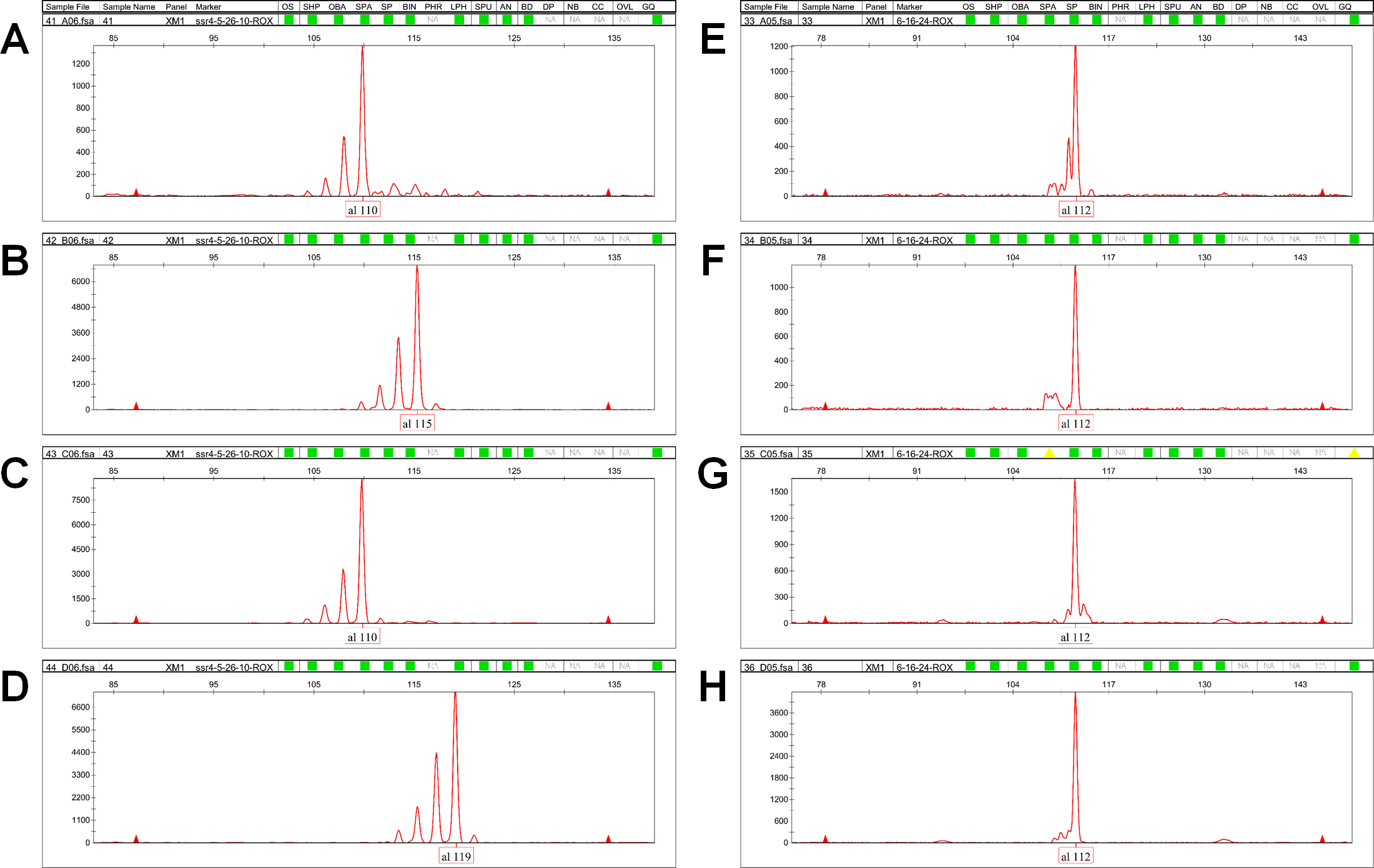

Supplement: Supplementary file 2 — Supplementary Figure 2. [file 41598_2023_29716_MOESM2_ESM.tif]
