# Supplementary material for: Genetic diversity between local landraces and current breeding lines of pepper in China
Source: Sci Rep. 2023 Mar 11;13:4058. doi: 10.1038/s41598-023-29716-4 (PMC10008637; doi:10.1038/s41598-023-29716-4)
Supplement: Supplementary file 5 — Supplementary Legends. [file 41598_2023_29716_MOESM5_ESM.docx]

Supplementary Figure 1. Polymorphysis of simple sequence repeat (SSR) marker Epms331 and Epms391 in accession. A and E, GW142. B and F, GW225 . C and G, GW273. D and H, GW323.

Supplementary Figure 2. Polymorphysis of simple sequence repeat (SSR) marker Epms397 and Es350 in accession. A and E, B030. B and F, 035. C and G, B043. D and H, B095.
